# Pilots and athletes: Different concerns, similar concussion non-disclosure

**DOI:** 10.1371/journal.pone.0215030

**Published:** 2019-05-01

**Authors:** Craig A. Foster, Christopher D’Lauro, Brian R. Johnson

**Affiliations:** 1 Department of Behavioral Sciences and Leadership, United States Air Force Academy, Colorado Springs, Colorado, United States of America; 2 Center for Military Psychiatry and Neuroscience, Walter Reed Army Institute of Research, Silver Spring, Maryland, United States of America; Middlesex University, UNITED KINGDOM

## Abstract

**Objectives:**

Concussion non-disclosure research has focused almost exclusively on athletes. The focus on athletic populations has been sensible considering athletes’ demonstrated susceptibility to sustaining and concealing concussions. Nevertheless, the habitual use of athletic populations has allowed researchers and practitioners to omit the development of generalized perceived costs and perceived rewards as critical determinants of concussion self-disclosure. We hypothesized that perceiving concussion disclosure as generally more costly than rewarding would predict negative attitudes towards disclosure and decreased intent to disclose. We also hypothesized that generalized perceived costs and rewards could explain concussion non-disclosure in different populations, athletes and future pilots specifically, even when those populations perceive concussion self-disclosure as costly for different specific reasons.

**Methods:**

We examined concussion disclosure using 2,504 cadets at the United States Air Force Academy. Cadets completed anonymous surveys assessing their intention to self-disclose undiagnosed concussions (*Anticipated Concussion Disclosure*) as well as several variables potentially related to concussion self-disclosure: perceived cost, perceived reward, personal identity, attitudes, normative behavior, social support, and self-efficacy.

**Results:**

The results demonstrate that concussion non-disclosure develops when a population perceives disclosure as more costly (i.e. directly or emotionally) and less rewarding. *Perceived Cost* and *Perceived Reward* variables alone accounted for 50% of the variance in *Anticipated Conclusion Disclosure* (*Adjusted R*^*2*^ *=* 0.50, *F*(2,2312) = 1,145.31, *p* < 0.001). As expected, *Anticipated Conclusion Disclosure* developed for different reasons within different sub-populations. Consistent with existing research, cadet intercollegiate athletes reported being primarily concerned that concussion self-disclosure would cause them to miss practice or game time (*t* (736.7) = 14.20, *p* < .001, *Cohen’s d* = 0.96). In contrast, cadet future pilots reported being primarily concerned that concussion self-disclosure would have negative United States Air Force career repercussions (*t* (1828) = 10.25, *p* < .001, *Cohen’s d* = 0.50).

**Conclusions:**

These results suggest that cultures of concussion non-disclosure can develop in any population where disclosure is perceived as having undesirable consequences, not just athletic populations. Concussion researchers and practitioners should devote more attention to the perceived cost-benefit structures that create concussion non-disclosure to address this crucial public health issue more effectively.

## Introduction

Public and scientific interest in concussions has grown rapidly over the past decade. Concussions have now been linked with numerous long-term health sequelae including increased risk of depression [1], neurodegeneration [2], suicide [3], and Chronic Traumatic Encephalopathy [4], among others. The Centers for Disease Control and Prevention (CDC) estimates that up to 3.8 million concussions occur per year in the United States [5]. Concussions can be treated, but a proper treatment plan requires patients to disclose their symptoms accurately [6]. Unfortunately, anywhere from 30.5%-69% of concussions go unreported [7, 8, 9, 10, 11], meaning many individuals lose the benefits of treatment [12].

Dozens of research studies have demonstrated that concussion non-disclosure is a pervasive problem in athletic populations [13, 14]. This literature has also identified several important contributors to concussion non-disclosure. In the personal domain, athletes conceal concussions for reasons such as not wanting to let down teammates [15] and believing that disclosure conflicts with their identity as an athlete [15, 16, 17]. Athletes also conceal concussions when they believe that the surrounding culture is not supportive of disclosure [18, 19]. Further, research has detailed a number of sport—and competition—related reasons why athletes might not disclose, including a lack of symptom knowledge [9, 20], fear of missing future competitions [9], not deeming a concussion serious enough to stop playing [8, 9, 7, 21], and expectations of playing injured [15].

This research, while informative, could expand in two important and interconnected ways. First, concussion non-disclosure research has examined athletic populations almost exclusively. The concern about athletes’ well-being is sensible and laudable. Sports medicine researchers have access to athletes as a research population, and learning more about their reluctance to disclose concussions can improve clinical practice. Nevertheless, the continued work with athlete populations might inadvertently frame concussion concealment as an athlete problem rather than a broader human problem. Furthermore, by expanding concussion self-disclosure research beyond the athlete domain, it forces one to consider the broader theoretical circumstances that can explain cultures of non-disclosure across different populations. In particular, we believe that cultures of concussion non-disclosure develop when any population develops a general belief that disclosure would be more costly than it is rewarding. The development of generalized perceived rewards and costs has not been pressing in the athletic domain because the specific reasons that cause athlete non-disclosure are relatively consistent (e.g., missing playing time, letting down teammates and coaches). These athlete-specific costs do not necessarily translate to other sub-populations that might have different specific anxieties about concussion self-disclosure, but the same general perception that disclosure could be more costly than rewarding.

Indeed, one can understand the importance of perceived costs and rewards by considering concussion disclosure as a specific type of social dilemma [22]. Individuals should generally be inclined to disclose their concussions and receive beneficial medical care. Concussion disclosure only becomes a social problem when individuals perceive disclosure as being costly [12, 23, 24]. For athletes, these perceived costs might be direct, such as being forced to miss competitions [25], but they also might be emotional, such as behaving in ways that feel inconsistent with personal identity [26] or losing status with close others [15]. Athletes might also have additional specific concerns about disclosure that are not captured by existing research, or their perception that disclosure is costly could be vague and not tied to any specific concerns. Accordingly, athletes often experience a tension between the perceived need to disclose their concussions and the potential consequences of doing so.

Non-athlete populations might experience this same type of dilemma, but for different reasons. For instance, non-athlete populations might conceal concussions because they associate concussion disclosure with losing income rather than playing time. In such a case, the specific concerns that athletes typically have would not predict concussion non-disclosure in this new population. Yet, both populations would have specific costs that create general perceptions of concussion disclosure being detrimental which, in turn, create negative attitudes towards disclosure and reduce disclosure itself. Put more broadly, both populations could develop cultures of concussion non-disclosure, even while the specific reasons causing the cultures of non-disclosure differ.

To examine this possibility, we conducted an extensive study at the United States Air Force Academy (USAFA). This study allowed us to extend concussion self-disclosure research beyond athlete populations and into a military and professional domain. By doing so, we could examine whether cultures of concussion non-disclosure develop for different reasons than those revealed by athlete populations and thereby highlight the importance of generalized perceived costs and rewards. Our large sample size also provided the statistical power to examine perceived costs and rewards (specific and generalized) along with additional variables identified as important in the concussion disclosure literature. We developed a brief hypothetical concussion scenario which allowed us to examine intent to disclose concussions across all USAFA cadets, not just those who are willing to disclose concussions.

We developed four general hypotheses, each of which examined generalized perceived costs and rewards as critical variables that can explain concussion non-disclosure across different populations. To begin, we examined whether generalized perceived costs and rewards would predict negative outcomes associated with concussion self-disclosure:

**Hypothesis 1a**: Attitudes about concussion self-disclosure will associate negatively with generalized perceived costs and positively with generalized perceived rewards (all participants).

**Hypothesis 1b**: Intent to self-disclose concussions will associate negatively with generalized perceived costs and positively with generalized perceived rewards (all participants).

We then used intercollegiate athlete and future pilot subpopulations to examine whether these subpopulations were vulnerable to concussion non-disclosure and, if so, whether generalized perceived costs and rewards would mediate the link between sub-population status (yes versus no) and concussion non-disclosure:

**Hypothesis 2a:** Generalized perceived costs and generalized perceived rewards will mediate any negative relationship between *intercollegiate athlete status* and attitudes about concussion disclosure.

**Hypothesis 2b**: Generalized perceived costs and generalized perceived rewards will mediate any negative relationship between *intercollegiate athlete status* and intent to self-disclose concussions.

**Hypothesis 3a:** Generalized perceived costs and generalized perceived rewards will mediate any negative relationship between *future pilot status* and attitudes about concussion disclosure.

**Hypothesis 3b:** Generalized perceived costs and generalized perceived rewards will mediate any negative relationship between *future pilot status* and intent to self-disclose concussions.

Finally, we examined whether intercollegiate athlete and future pilot sub-populations differ in their specific concerns about concussion non-disclosure even while they exhibit similar reluctance to report concussions generally:

**Hypothesis 4:** Intercollegiate athletes and future pilots will identify different primary specific concerns about concussion self-disclosure.

## Methods

### Participants and procedure

Participants were 2,504 USAFA cadets (573 Female; 1,815 Male; 116 did not respond) at the USAFA. The USAFA is a federal military academy where cadets receive a Bachelor of Science degree upon graduation. Graduates typically incur a five-year service commitment to the United States Air Force (USAF). Graduates who complete pilot training typically incur a longer service commitment. We initially included all participants who responded to at least one of the non-demographic items (*N* = 2,531) listed below. We then omitted 27 participants who responded with one number (e.g. 9) for all 12 of the items pertaining to general and specific perceived rewards and costs (which were on the survey front page) or with one number for all 17 of the *Identity*, *Attitude*, *Subjective Norms*, *Social Support*, and *Self-Efficacy* items (which were on the second page of the survey).

We included this concussion scenario in a broader survey that we incorporated into the Concussion, Assessment, Research, and Education (CARE) baseline administration at the USAFA [27]. Participants completed surveys when time permitted before, during, or immediately after the process for concussion baseline testing just before and during the academic summer of 2015. Participation was voluntary. We kept the survey brief to encourage participation. The survey was limited to the front and back of one 8.5-by-11-inch sheet of paper. This protocol was approved and declared exempt by the USAFA Institutional Review Board.

### Survey

The survey began with instructions informing participants that the survey examined cadets’ feelings about reporting concussions. The instructions indicated that participants would not be asked to provide any information that could identify them personally and encouraged participants to therefore feel comfortable about responding honestly.

Participants were then presented with a concussion scenario: **‘**Imagine that you are confident that you suffered a concussion and you believe that you are experiencing concussion-related symptoms. Despite these symptoms, you are confident that nobody knows about your concussion. Thus, if you kept this information to yourself, you are confident that nobody at the USAFA would know about your concussion’. This scenario was followed by the *Anticipated Concussion Disclosure* (*ACD*) question: ‘How likely is it that you would report the potential concussion to medical staff (e.g. cadet clinic, athletic trainers, athletic coaches, EMTs)?’ Participants could respond by circling an answer on a scale ranging from 1 (*Very Unlikely to Report Concussion*) to 9 (*Very Likely to Report Concussion*) (We changed these scale anchors and the parallel anchors associated with the *Average Cadet* measure in an attempt to minimize participants overlooking these measures; approximately 19% of participants had “*Very Unlikely*” and “*Very Likely*” as scale anchors). We developed this scenario-based *ACD* measure to provide a uniform method for measuring intent to disclose across all participants and preclude the variance that would accompany participants imagining their own particular circumstances for concussion disclosure in an open-ended behavioral intent measure.

The survey then presented several items and a corresponding scale that ranged from 1 (*Strongly Disagree*) to 9 (*Strongly Agree*). The first set of items examined rewards and costs associated with disclosure. All 12 items began with ‘Reporting the concussion to medical staff would…’ and ended with (1) be costly to me; (2) create negative outcomes for me; (3) be beneficial to me; (4) create positive outcomes for me; (5) create negative outcomes for me in my USAF career; (6) cause me to miss intercollegiate practice or game time; (7) prevent me from participating in airfield activities; (8) prevent me from being commissioned; (9) result in my disenrollment from USAFA; (10) result in my falling behind in class; (11) simply be a hassle; (12) get me out of things that are annoying. We averaged the first two items to create a measure of *Perceived Cost* (Cronbach’s alpha = 0.87; all reported alphas were non-standardized) and the third and fourth items to create a measure of *Perceived Reward* (Cronbach’s alpha = 0.88).

Participants were then provided a scale ranging from 1 (Strongly Disagree) to 9 (Strongly Agree) to use in responding to several items. The first seven items examined whether a particular attribute was part of their identity (tough; conscientiousness; self-reliant; an athlete; a warrior; a military professional; importance of becoming a military pilot). We developed these items based on existing research demonstrating the influence of athletes identifying as tough [28], conscientious [13], or as athletes generally [16] and the potential relevance of cadets identifying as warriors and as military pilots.

These identity-based items were followed by several items based on the theory of planned behavior, a well-established theory about the predictors of human behavior which has been used extensively to examine concussion disclosure [29, 30, 31, 32]. Two items measured the degree to which participants had a positive or negative attitude about concussion self-disclosure (negative attitude item was reverse-scored; items were averaged to create a measure of *Attitude*; Cronbach’s alpha = 0.88). Two items measured perceptions that other cadets would be supportive or unsupportive towards their disclosing a concussion (unsupportive item was reverse-scored; items were averaged to create a measure of *Subjective Norms*; Cronbach’s alpha = 0.87). Two items measured whether cadets who were important to them would be supportive or unsupportive towards their disclosing a concussion (unsupportive item was reverse-scored; items were averaged to create a measure of *Social Support*; Cronbach’s alpha = 0.82).

This section of the survey concluded with four self-efficacy items adapted from items used in previous concussion-disclosure research [30]. These items assessed (1) confidence in recognizing symptoms, (2) reporting even when doing so is undesirable, (3) reporting when symptoms are not all that bad, and (4) reporting even when unsure about truly having a concussion (items were averaged to create a measure of *Self-Efficacy*; Cronbach’s alpha = .84).

Next, participants were asked to indicate the likelihood that the *Average Cadet* would disclose a concussion in the provided scenario; this item was followed by its own scale ranging from 1 (*Very Unlikely to Report Concussion*) to 9 (*Very Likely to Report Concussion*).

The survey concluded by prompting participants to indicate *Sex* (Male, Female), graduating *Class Year* (2016, 2017, 2018, 2019), *Intercollegiate Athlete* status (Yes, No), desire to become a *Future Pilot* (Yes, No), *Pilot Qualification* status (Yes, No), participation in *Intercollegiate Football* (Yes, No), and participation in a *High-Impact (HI) Intercollegiate Sport* (i.e. ice hockey, soccer, basketball, wrestling, or lacrosse; Yes, No). Cadets at the USAFA are designated as having intercollegiate status for administrative purposes. It is possible that a small number of cadets who were not traditional intercollegiate athletes (e.g. team managers) might have identified as having intercollegiate status. The inclusion of these cadets would possibly reduce the apparent differences between intercollegiate athletes and non-intercollegiate athletes.

## Results

### Anticipated concussion disclosure and descriptive statistics

Table 1 shows the descriptive statistics for *ACD* and the other variables. Participants reported considerable variation in terms of *ACD*. Participants reported, on average, that they anticipated disclosing their concussions in the context of the provided scenario. Of the participants who responded to the *ACD* scenario, the majority provided responses above the scale midpoint (*n* = 1,503; 64.4%) indicating a modest or strong intention to disclose. Participants’ modal response indicated the highest likelihood of *ACD* (i.e. 9; *n* = 468; 20.1%) followed by a moderate *ACD* (i.e. 7; *n* = 434; 18.6%). At the same time, many participants responded below the scale midpoint indicating a reluctance to disclose their concussions (*n* = 609; 26.1%). Some participants reported the lowest possible level of *ACD* (i.e. 1; *n* = 155; 6.6%).

**Table 1:** Descriptive statistics.

Variable	*n*	*M(SD)*	Median	Mode
ACD	2,332	6.07 (2.47)	7.00	9
Perceived Cost	2,476	4.41 (2.51)	4.50	1
Perceived Reward	2,472	5.99 (2.20)	6.00	9
Attitude	2,404	5.94 (2.20)	6.00	5
Subjective Norms	2,399	6.18 (2.06)	6.50	5
Social Support	2,397	7.19 (1.74)	7.50	9
Self-Efficacy	2,393	5.73 (1.85)	5.75	7
Tough	2,414	6.55 (1.91)	7.00	7
Conscientious	2,390	6.90 (1.64)	7.00	7
Self-Reliant	2,415	7.43 (1.44)	8.00	8
Athlete	2,415	6.64 (2.20)	7.00	9
Warrior	2,414	6.74 (1.89)	7.00	7
Military Professional	2,411	6.95 (1.82)	7.00	9
Military Pilot Importance	2,411	5.60 (3.08)	6.00	9
Negative Career Outcomes	2,471	5.24 (2.62)	5.00	5
Miss Practice/Game Time	2,318	5.98 (3.01)	7.00	9
Miss Airfield Activities	2,413	6.71 (2.54)	7.00	9
Lose Commission	2,464	3.77 (2.42)	3.00	1
Disenrollment	2,468	2.79 (2.16)	2.00	1
Falling Behind in Class	2,469	4.50 (2.54)	5.00	1
Reporting is a Hassle	2,467	4.76 (2.47)	5.00	5
Avoid Annoying Tasks	2,463	4.08 (2.37)	4.00	1
Average Cadet	2,039	4.94 (1.77)	5.00	6

Table 1 shows the descriptive statistics–mean, standard deviation, median, and mode for each item–as well as the number of participants who completed each item. All variables had the lowest possible Minimum (1) and the highest possible Maximum (9). ACD = Anticipated Concussion Disclosure

### Predicting anticipated concussion disclosure

We examined the relative importance of variables related to perceived costs and rewards, attitudes, perceptions of how others’ support concussion self-reporting, and identity for predicting *ACD*. Specifically, we conducted a multivariate linear regression using simultaneously all items related to participant *Identity*, *Perceived Cost*, *Perceived Reward*, *Attitude*, *Subjective Norms*, *Social Support*, and *Self-Efficacy* to examine broadly and simultaneously their relative importance in predicting *ACD*. Table 2 shows that identity played a relatively minor role. Variables related to the situational context were much stronger predictors of *ACD*. In particular, *Perceived Cost* predicted reduced *ACD*, whereas *Perceived Reward*, positive *Attitude*, and *Self-Efficacy* all predicted greater *ACD*. The overall model predicted almost 60% of the variance in *ACD* (*Adjusted R*^*2*^ = 0.59, *F*(13,2173) = 247.21, *p* < 0.001).

**Table 2:** Regression using the following variables as simultaneous predictors of anticipated concussion disclosure.

Variable	ß	*t*	*p*
Percieved Cost	-0.164	-8.40	< 0.001
Perceived Reward	0.273	13.29	< 0.001
Attitude	0.364	17.70	< 0.001
Subjective Norms	-0.022	-1.22	= 0.222
Social Support	0.021	1.24	= 0.216
Self-Efficacy	0.114	7.24	< 0.001
Tough	-0.004	-0.21	= 0.832
Conscientious	0.049	3.29	= 0.001
Self-Reliant	-0.020	-1.31	= 0.191
Athlete	-0.004	-0.22	= 0.825
Warrior	-0.021	-1.06	= 0.290
Military Professional	-0.006	-0.36	= 0.719
Military Pilot Importance	0.003	0.22	= 0.829

Table 2 shows the beta weight, *t* value, and *p* value for each measure used to predict *Anticipated Concussion Disclosure* in a simultaneous regression.

We examined *Hypothesis 1* specifically by conducting a multivariate linear regression using *Perceived Cost and Perceived Reward* to predict *Attitude* and a parallel regression to predict *ACD*. We omitted *Self-Efficacy* from further analysis because of its theoretical overlap with our *ACD* measure. Consistent with *Hypothesis 1a*, *Perceived Cost* (ß = -0.31, *t* = -15.37, *p* < .001) and *Perceived Reward* (ß = 0.44, *t* = 21.82, *p* < .001) accounted for 47% of the variance in *Attitude* (*Adjusted R*^*2*^ = 0.47, *F*(2,2378) = 1,047.39, *p* < 0.001). Consistent with *Hypothesis 1b*, *Perceived Cost* (ß = -0.29, *t* = -14.82, *p* < .001) and *Perceived Reward* (ß = 0.48, *t* = 24.37, *p* < .001) accounted for 50% of the variance in *ACD* (*Adjusted R*^*2*^ = 0.50, *F*(2,2312) = 1,145.31, *p* < 0.001).

Next, we examined the specific consequences that make concussion disclosure appear costly for participants generally. We used all seven specific costs and the one specific reward (i.e., getting out of things that are annoying) associated with concussion disclosure as simultaneous predictors and *Perceived Cost* as the outcome variable (*Adjusted R*^*2*^ = 0.47, *F*(8,2280) = 258.62, *p* < 0.001). We did not conduct this analysis using *Perceived Reward* (i.e., the generalized *Perceived Reward* measure) because all but one of the specific items were framed around costs and *Perceived Cost* correlated strongly with *Perceived Reward* (*n* = 2,470, *r* = -0.66, *p* < 0.001). Table 3 shows that the belief that concussion disclosure would create negative USAF career outcomes was the strongest predictor of *Perceived Cost*. Disclosure being ‘a hassle’ also was an important predictor of *Perceived Cost*. Finally, missing practice or game time and potential disenrollment from USAFA were weaker but statistically significant predictors.

**Table 3:** Regression using specific costs and reward as simultaneous predictors of perceived cost.

Specific Cost/Reward	ß	*t*	*p*
Negative Career Outcomes	0.562	30.02	< 0.001
Miss Practice/Game Time	0.057	3.26	= 0.001
Miss Airfield Activities	0.008	0.46	= 0.644
Lose Commission	-0.002	-0.06	= 0.948
Disenrollment	0.067	2.94	= 0.003
Falling Behind in Class	0.001	0.07	= 0.940
Reporting is a Hassle	0.185	10.71	< 0.001
Avoid Annoying Tasks	-0.024	-1.53	= 0.127

Table 3 shows the beta weight, *t* value, and *p* value for each specific cost and the one reward used to predict *Perceived Cost in a simultaneous regression*.

### Intercollegiate athletes versus non-intercollegiate athletes

We developed a particular analytic strategy to test further our general belief that perceived costs and rewards contribute substantially to negative attitudes about concussion disclosure and to concussion non-disclosure. We began by examining whether *Attitude* and *ACD* varied as a function of being an *Intercollegiate Athlete* (Yes, No) and *Class Year* (First-Year through Fourth-Year) while including only participants who reported explicitly that they did not want to be *Future Pilots* (included *n* = 886). We adopted this strategy for three main reasons. First, a fundamental component of the present research was to examine whether perceived costs and rewards create cultures of concussion non-disclosure; accordingly, we wanted to examine whether USAFA athletes reduce concussion self-disclosure due to concerns about missing practice or game time, like civilian athletes do, and then examine whether future pilots reduce concussion self-disclosure for different reasons. Second, the proportion of cadets reporting that they wanted to become pilots was smaller for cadets who reported being an *Intercollegiate Athlete* (*n* = 267 of 512, 52.1%) than it was for cadets who reported not being an *Intercollegiate Athlete* (*n* = 1,206 of 1,847, 65.3%); we wanted to remove this subtle form of confounding. Third, analyses using all participants indicated that *Class Year* was an important component of concussion disclosure with cadets reporting lower *ACD* as they were closer to graduation (*F*(3,2234) = 40.18, *p* < 0.001; First-Year, *M* = 6.77, *SD* = 2.02, *n* = 820; Second-Year, *M* = 5.96, *SD* = 2.46, *n* = 555; Third-Year, *M* = 5.75, *SD* = 2.59, *n* = 372; Fourth-Year, *M* = 5.36, *SD* = 2.70, *n* = 491).

**Fig 1:**
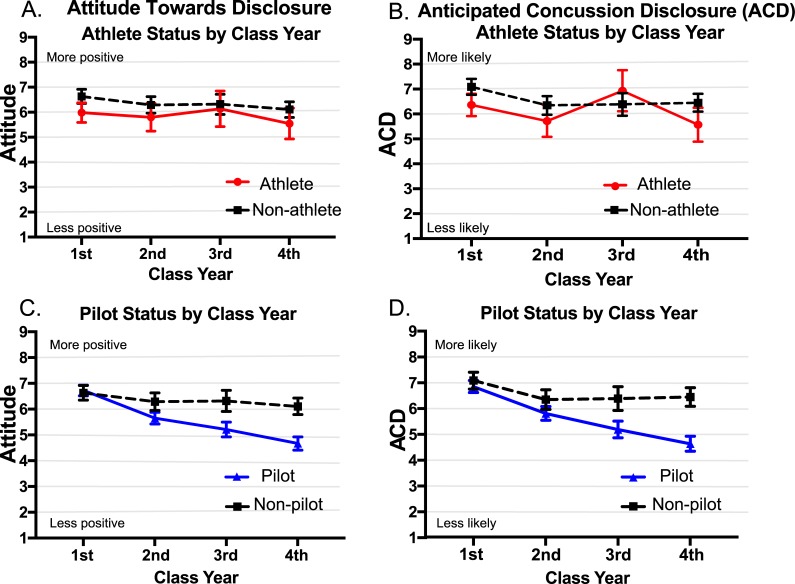
(a) Shows Attitude towards disclosure and (b) ACD (Mean and 95% CI error bars for all figures) for Athletes and Non-athletes across four class years. Future pilots were excluded from analyses. (c) Shows Attitude towards disclosure and (d) ACD for future Pilots and Non-pilots across four class years. Intercollegiate athletes were excluded from analyses.

We began by conducting a 2 X 4 ANOVA using *Intercollegiate Athlete* (Yes, No) and *Class Year* (First-Year through Fourth-Year) as between-groups factors and *Attitude* as the dependent variable. The obtained results revealed no significant *Intercollegiate Athlete* by *Class Year* interaction (*F*(3,869) = 0.32, *p* = 0.810) and no significant effect for *Class Year* (*F*(3,869) = 1.86, *p* = 0.136), but there was a main effect for *Intercollegiate Athlete* (*F*(1,869) = 7.59, *p* = 0.006). Fig 1A shows that cadets who reported being intercollegiate athletes, relative to those who did not, reported less positive attitudes about concussion disclosure. We conducted the same 2 x 4 ANOVA using *ACD* as the dependent variable. The obtained results revealed a marginally significant *Intercollegiate Athlete* by *Class Year* interaction (*F*(3,818) = 2.13, *p* = 0.095), a main effect for *Intercollegiate Athlete* (*F*(1,818) = 4.69, *p* = 0.031), and a main effect for *Class Year* (*F*(3,818) = 4.75, *p* = 0.003). Fig 1B shows that cadets who reported being intercollegiate athletes, relative to non-intercollegiate athletes, generally reported lower *ACD* (with an unexpected exception among cadets in their third year) and cadets reported lower *ACD* the closer they were to graduation.

To examine *Hypothesis 2* –that perceived costs and rewards mediate a relationship between intercollegiate athlete status and negative outcomes related to concussion self-disclosure–we conducted ANCOVAs by replicating the previous analyses while including *Perceived Cost* and *Perceived Reward* as covariates. The ANCOVA using *Attitude* as a dependent variable revealed no *Intercollegiate Athlete* by *Class Year interaction* (*F*(3,861) = 0.11, *p* = 0.953) and no *Class Year* main effect (*F*(3,861) = 0.37, *p* = 0.773). The *Intercollegiate Athlete* main effect decreased, but was still statistically significant (*F*(1,861) = 3.97, *p* = .047). Both *Perceived Cost* (*F*(1,861) = 76.82, *p* < 0.001) and *Perceived Reward* (*F*(1,861) = 176.43, *p* < 0.001) were significant covariates. These results suggest that *Perceived Cost* and *Perceived Reward* partially mediate the influence of *Intercollegiate Athlete* on *Attitude*. The ANCOVA using *ACD* as a dependent variable revealed no significant *Intercollegiate Athlete* by *Class Year* interaction (*F*(3,814) = 1.52, *p* = 0.209) and no *Intercollegiate Athlete* main effect (*F*(1,814) = 1.35, *p* = 0.246), but the *Class Year* main effect (*F*(3,814) = 4.82, *p* = 0.002) remained. Both the *Perceived Cost* covariate (*F*(1,814) = 45.99, *p* < 0.001) and *Perceived Reward* covariate (*F*(1,814) = 237.23, *p* < 0.001) were statistically significant. These results suggest that *Perceived Cost* and *Perceived Reward* mediate the influence of *Intercollegiate Athlete* on *ACD*.

We then examined how intercollegiate athletes perceive concussion self-disclosure relative to non-athletes. We conducted a series of *t* tests using *Perceived Cost*, *Perceived Reward*, the seven costs, and the one reward as dependent variables and *Intercollegiate Athlete* (Yes, No) as the categorical variable. Table 4 shows that USAFA intercollegiate athletes, relative to non-intercollegiate athletes, reported greater generalized *Perceived Cost* associated with concussion disclosure and likely conceal concussions for reasons similar to their civilian athlete counterparts. Cadets who were intercollegiate athletes, relative to those who were not, reported much stronger agreement that concussion disclosure would interfere with practice or game time. The remaining differences between intercollegiate athletes and non-intercollegiate athletes were much smaller by comparison. Intercollegiate athletes reported stronger agreement that concussion disclosure would cause them to miss airfield activities (e.g. flying, parachuting) and marginally stronger agreement that disclosure would cause them to fall behind in class. Intercollegiate athletes also reported reduced agreement that concussion disclosure would lead to disenrollment.

**Table 4:** Perceived cost and reward as a function of intercollegiate athlete status.

	Athlete (Non-Pilot)		Non-Athlete (Non-Pilot)				
Variable	*n*	*M (SD)*	*n*	*M (SD)*	*t*	*p*	*d*
Perceived Cost	245	4.21 (2.37)	636	3.62 (2.26)	3.42	< 0.001	0.25
Perceived Reward	245	6.22 (2.05)	635	6.44 (2.03)	-1.46	= 0.146	-0.11
Negative Career Outcomes	243	4.37 (2.40)	635	4.56 (2.50)	-1.03	= 0.302	-0.08
Miss Practice/Game Time	245	7.84 (1.87)	603	5.35 (3.15)	14.2	< 0.001^a^	0.96
Miss Airfield Activities	243	6.43 (2.63)	611	5.88 (2.89)	2.68	= 0.008^a^	0.20
Lose Commission	244	3.57 (2.38)	636	3.52 (2.40)	0.28	= 0.782	0.02
Disenrollment	245	2.47 (1.90)	635	2.79 (2.20)	-2.15	= 0.032^a^	-0.16
Falling Behind in Class	245	4.77 (2.47)	636	4.41 (2.57)	1.89	= 0.058	0.14
Reporting is a Hassle	245	4.84 (2.40)	634	4.64 (2.48)	1.11	= 0.267	0.08
Avoid Annoying Tasks	244	4.30 (2.30)	635	4.05 (2.40)	1.37	= 0.171	0.11

Table 4 shows the *t* value, *p* value and *Cohen’s d* for each measure in the comparisons between *Intercollegiate Athletes* and *Non-Intercollegiate Athletes* (*Future Pilots* excluded). When the groups showed unequal variances, we reported results where equal variances were not assumed. These cases are marked with an ^a^.

When examining these differences, it is important to remember that they compare non-pilot *Intercollegiate Athletes* to non-pilot non-*Intercollegiate Athletes*. We were nonetheless surprised that the *Intercollegiate Athletes* reported stronger agreement that concussions would interfere with airfield activities. One possibility is that the item followed the missing practice and game-time item and an anchoring effect raised *Intercollegiate Athletes’* responses to the airfield activities item without providing the same effect for the non-*Intercollegiate Athletes’* responses. It might also be that for some unexpected reason non-pilot non-Intercollegiate athletes exhibit low levels of engagement with the airfield. For example, the USAF, like the USAFA has a salient pilot/non-pilot culture, which might make non-pilot non-athletes more likely to view those activities primarily as things that future pilots do.

### Future pilots versus non-pilots

We conducted parallel analyses to examine future pilots versus non-pilots. We were particularly interested in future pilots because we believed that they were susceptible to concussion non-disclosure due to concerns about losing their qualifications to serve as pilots in the USAF. We examined future pilots versus non-pilots including only cadets who reported explicitly that they were not intercollegiate athletes (included *n* = 1,871). We conducted a 2 X 4 ANOVA using *Future Pilot* (Yes, No) and *Class Year* as between-groups factors and *Attitude* as the dependent variable. The results revealed a *Future Pilot* by *Class Year* interaction (*F*(3, 1828) = 11.48, *p* < 0.001), a main effect for *Future Pilot* (*F*(1, 1828) = 51.48, *p* < 0.001), and a main effect for *Class Year* (*F*(3, 1828) = 31.58, *p* < 0.001). Fig 1C shows that cadets who planned to become pilots, relative to the remaining cadets, reported more negative attitudes about concussion disclosure as they neared graduation. A parallel 2 x 4 ANOVA using *ACD* as the dependent variable revealed a *Future Pilot* by *Class Year* interaction (*F*(3, 1727) = 9.62, *p* < 0.001), a main effect for *Future Pilot* (*F*(1, 1727) = 60.19, *p* < 0.001), and a main effect for *Class Year* (*F*(3, 1727) = 32.19, *p* < 0.001). Fig 1D shows that cadets who wanted to become pilots, relative to the remaining cadets, enter the Academy with similar *ACD* but they were increasingly reluctant to disclose concussions as they neared graduation.

We then examined *Hypothesis 3* –that perceived costs and rewards mediate a relationship between future pilot status and negative outcomes related to concussion self-disclosure. We conducted ANCOVAs by replicating the previous analyses while including *Perceived Cost* and *Perceived Reward* as covariates, again to test *Perceived Cost* and *Perceived Reward* as potential mediators. The ANCOVA using *Attitude* as the dependent variable revealed the *Future Pilot* main effect (*F*(1, 1810) = 0.61, *p* = 0.436) was no longer significant, whereas *Perceived Cost* (*F*(1, 1810) = 178.57, *p* < 0.001), *Perceived Reward* (*F*(1, 1810) = 365.01, *p* < 0.001), and the main effect for *Class Year* (*F*(3, 1810) = 7.44, *p* < 0.001) were statistically significant. Interestingly, this ANCOVA revealed a crossover *Future Pilot* by *Class Year* interaction (*F*(3, 1810) = 3.37, *p* = 0.018); this interaction appears to be due to future pilots having more positive attitudes about concussion disclosure than non-pilots when both groups were in their first year (First-Year Future Pilots: Estimated Marginal Mean = 6.35; 95%CI = 6.20 to 6.51; First-Year Non-Pilots: Estimated Marginal Mean = 6.05; 95%CI = 5.83 to 6.27) but more negative attitudes than non-pilots when both groups were in their fourth and final year (Fourth-Year Future Pilots: Estimated Marginal Mean = 5.62; 95%CI = 5.42 to 5.81; Fourth-Year Non-Pilots: Estimated Marginal Mean = 5.95; 95%CI = 5.71 to 6.19) after including *Perceived Cost* and *Perceived Reward* as covariates. Similarly, the ANCOVA using *ACD* as the dependent variable revealed that the *Future Pilot* by *Class Year* interaction (*F*(3, 1722) = 2.53 *p* = 0.055) and the *Future Pilot* main effect (*F*(1, 1722) = 1.49, *p* = 0.223) were no longer statistically significant, whereas *Perceived Cost* (*F*(1, 1722) = 161.73, *p* < 0.001), *Perceived Reward* (*F*(1, 1722) = 421.55, *p* < 0.001), and the main effect for *Class Year* (*F*(3, 1722) = 10.57, *p* < 0.001) were statistically significant. These results indicate that *Perceived Cost* and *Perceived Reward* mediated the influence of *Future Pilot* on *ACD*. These results indicate that, once the variance of *Perceived Cost* and *Perceived Reward* is accounted for, *Future Pilots* progress from having more positive attitudes to more negative attitudes than non-pilots and that *Perceived Cost* and *Perceived Reward* mediate the main effect of *Future Pilot* on *ACD*.

Finally, we examined how future pilots perceive concussion self-disclosure relative to non-pilots. This test, combined with the earlier parallel results using intercollegiate athletes, allowed us to test *Hypothesis 4* –that these two populations report different specific concerns associated with concussion self-disclosure, even while they both exhibit similar generalized concerns about concussion self-reporting. We conducted a series of *t* tests using *Perceived Cost*, *Perceived Reward*, the seven costs, and the one reward as dependent variables and *Future Pilot* (Yes, No) as the categorical variable. Table 5 shows that cadets who wanted to become pilots, relative to those who did not, perceived concussion disclosure as more costly and less rewarding. Table 5 also shows that future pilots, relative to non-pilots, reported stronger agreement that concussion disclosure would create negative career outcomes and cause them to miss airfield activities. Future pilots also reported, relative to non-pilots, stronger agreement that concussion disclosure would cause them to lose their commission and would be a hassle. Consistent with *Hypothesis 4*, the emergence of negative career outcomes and missing airfield activities as standout specific concerns about concussion self-disclosure was in contrast to the athlete population who reported missing practice or game time as the standout specific concern about concussion self-disclosure, like their civilian counterparts (see Table 4).

**Table 5:** Perceived costs and reward as a function of future pilot status.

	Pilot (Non-Athlete)		Non-Pilot (Non-Athlete)				
Variable	*n*	*M (SD)*	*n*	*M (SD)*	*t*	*p*	*d*
Costs	1,196	4.81 (2.54)	636	3.62 (2.26)	10.19	< 0.001^a^	0.49
Rewards	1,197	5.75 (2.24)	635	6.44 (2.03)	-6.74	< 0.001^a^	-0.34
Negative Career Outcomes	1,195	5.83 (2.55)	635	4.56 (2.50)	10.25	< 0.001	0.50
Miss Practice/Game Time	1,087	5.46 (3.03)	603	5.35 (3.15)	0.68	= 0.494^a^	0.04
Miss Airfield Activities	1,175	7.16 (2.23)	611	5.88 (2.89)	9.59	< 0.001^a^	0.50
Lose Commission	1,194	4.01 (2.46)	636	3.52 (2.40)	4.14	< 0.001	0.20
Disenrollment	1,195	2.92 (2.20)	635	2.79 (2.20)	1.21	= 0.225	0.06
Falling Behind in Class	1,194	4.48 (2.51)	636	4.41 (2.57)	0.55	= 0.586	0.03
Reporting is a Hassle	1,194	4.88 (2.46)	634	4.64 (2.48)	1.98	= 0.048	0.10
Avoid Annoying Tasks	1,189	4.01 (2.35)	635	4.05 (2.40)	-0.35	= 0.724	-0.02

Table 5 shows the *t* value, *p* value and *Cohen’s d* for each measure in the comparisons between *Future Pilots* and *Non-Future Pilots* (*Intercollegiate Athletes* excluded). When the groups showed unequal variance, we reported results where equal variances were not assumed. These cases are marked with an ^a^.

### Participant sex

Existing research suggests that males, relative to females, are generally more willing to lie [33, 34] and less willing to cooperate [35, 36]. Within sports medicine, research demonstrates that females might be more willing to endorse concussion symptoms in baseline testing [37] and that female athletes might be more likely to self-disclose [36, 38, 39, 40, 41, 42]. Accordingly, we felt it necessary to ensure that participant sex was not adversely influencing our results, particularly considering men were more likely to report wanting to become future pilots (68.3%, 1,222 of 1,790 respondents) than were women (44.6%, 251 of 563 total respondents; χ^2^ = 102.63, *p* < .001). Men were not more likely to report being intercollegiate athletes (22.0%, 398 of 1,810 respondents) than were women (21.3%, 121 of 569 total respondents; χ^2^ = 0.13, *p* = .72). An independent samples *t* test using *Participant Sex* (Men, Women) and *ACD* revealed that Men reported less *ACD* (*M* = 6.01, *SD* = 2.47, *n* = 1,695) than did women (*M* = 6.33, *SD* = 2.37, *n* = 534; *t*(2227) = -2.59, *p* = .010).

We then examined whether this effect persisted when accounting for *Future Pilot* status. We conducted a 2 x 2 ANOVA using *Participant Sex* (Men, Women) and *Future Pilot* (Yes, No) as between-groups factors and *ACD* as the outcome variable. The obtained results did not reveal a significant *Participant Sex* x *Future Pilot* interaction (*F*(1, 2190) = 1.21, *p* = .27) or *Participant Sex* main effect (*F*(1, 2190) = 2.50, *p* = .11), but did reveal a main effect for *Future Pilot* (*F*(1, 2190) = 28.33, *p* < .001) where participants reporting wanting to become pilots showed less *ACD* (*M* = 5.85, *SD* = 2.49, *n* = 1,373) relative to participants who did not (*M* = 6.49, *SD* = 2.33, *n* = 821). We believe these results demonstrate that participant sex was not likely to have a meaningful influence on the results obtained in the present research. Furthermore, these results also suggest that the perceived consequences associated with concussion self-disclosure for future pilots exerted a much stronger influence on concussion self-disclosure than did any influence associated with participant sex.

### Non-normality and anticipated concussion disclosure

Our primary dependent variable, *ACD*, had a skewness value of -0.59. To ensure that skew did not notably influence the obtained results, we performed a Blom transformation on *ACD* to create a new *Transformed ACD* variable. The *Transformed ACD* variable had a skewness value of -0.20. *Transformed ACD* had an almost perfect correlation with the original *ACD* variable (*r* = .987). To ensure that skew had no adverse influences on our results, we re-created our analyses using *ACD*, by replacing the original *ACD* variable with the *Transformed ACD* variable. These statistical recreations maintained the same pattern of statistically significant and non-significant results.

## Discussion

### Understanding concussion non-disclosure

We developed the present research to examine two important and interconnected issues in concussion self-disclosure. First, we wanted to evaluate whether substantial levels of concussion non-disclosure can occur in a non-athletic population–specifically a military population who might associate concussion disclosure with negative occupational consequences. Second, in so doing, we wanted to understand the fundamental role that generalized perceived costs and rewards play in creating concussion non-disclosure across different populations and within populations.

As expected, the obtained results demonstrated that non-athletic populations can be similarly susceptible to concussion non-disclosure and concussion non-disclosure might be a more widespread problem than has been demonstrated by research to date. Whereas cadets generally reported being favorably disposed to concussion disclosure, a notable portion of cadets (26.1%) reported that they were more likely to conceal their concussions than disclose them. The heightened reluctance to disclose concussions was evident in USAFA intercollegiate athletes relative to non-intercollegiate athletes. Nevertheless, the broad reluctance to disclose concussions at the USAFA appears to be created primarily by cadets who want to become pilots, a sub-population that was much larger than the sub-population of intercollegiate athletes. Future pilots reported decreased intent to disclose their concussions relative to non-future pilots, an effect that grew stronger as they neared graduation. Cadets reporting a desire to become pilots also reported that they perceived concussion disclosure to be costly, primarily due to potential USAF career repercussions and missing airfield activities.

The use of intercollegiate athlete and future pilot sub-populations motivated our emphasis on generalized perceived costs and rewards as fundamental contributors to concussion non-disclosure. We predicted, based on our broader promotion of concussion self-disclosure as part of a social dilemma, that generalized perceived costs and rewards could explain non-disclosure in both populations, even while each population revealed group-level differences in their specific concerns about self-disclosure. This general prediction was supported in different ways. We demonstrated that perceived costs and rewards were particularly important predictors of concussion disclosure, much more so than ‘athlete culture’ variables that are often narratively connected to non-disclosure [13, 14]. To begin, perceived costs and rewards predicted *ACD* far more strongly than did identity, subjective norms, and social support (variables commonly associated with athlete reluctance to disclose concussions) [13] [*Hypothesis 1*]. Furthermore, the obtained results indicated that the relationship between intercollegiate athlete status and negative outcomes associated with concussion disclosure (more negative attitudes and reduced *ACD*) could largely be explained by generalized perceived costs and rewards [*Hypothesis 2*]. Similarly, the obtained results indicated that the relationship between future pilot status and negative outcomes associated with concussion disclosure (more negative attitudes and reduced *ACD*) could largely be explained by generalized perceived costs and rewards [*Hypothesis 3*]. The similar explanatory power of generalized perceived costs and rewards for intercollegiate athletes and future pilots occurred even while intercollegiate athletes associated concussion disclosure primarily with the missing practice or game time as a specific cost–consistent with existing research into athlete populations [13, 14, 15, 16]–and future pilots associated concussion disclosure primarily with negative USAF career outcomes and missing airfield activities as specific costs [Hypothesis 4].

We believe that the evidence in favor of generalized perceived costs and rewards predicting concussion self-disclosure behavior highlights two fundamental ways of examining cultures of concussion non-disclosure. Researchers and practitioners could interpret existing research involving athlete concussion self-disclosure as indicating that athletes develop cultures that discourage concussion disclosure. This interpretation follows research indicating that athlete concussion disclosure is influenced by athlete identity [16], social support [43], and social norms [16, 44]. This perspective could be used to suggest that something about athletes and athlete culture causes athlete reluctance to disclose concussions [9, 21, 45]. The present research highlights a different perspective. The perceived cost-benefit structure surrounding concussion disclosure *generates* both the negative attitudes towards disclosure and non-disclosure behavior. Individuals in these environments discourage concussion disclosure due to the real or perceived costs associated with disclosure [18]. Put more broadly, environments where individuals have some reason to believe that concussion self-disclosure could be costly likely create cultures of concussion underreporting. These two perspectives could contribute to a vicious cycle. Perceived costs create cultures of non-disclosure which, in turn, reinforce the negative perceptions and attitudes around concussion disclosure. Cultures of concussion disclosure might function similarly even though they are generated by different types of perceived costs and rewards; athletes do not want to miss out on playing, but future pilots do not want to miss out on flying.

Further, the development of generalized perceived costs and rewards can help researchers overcome some of the limitations of survey research. Surveys must always balance between brevity and comprehensiveness in gathering information. A recent investigation [46] showed that concussed athletes had worse driving performance–poor enough to recommend they not drive. Participants then indicated that this recommendation would make them less likely to disclose a concussion. This research provides a specific cost that might reduce concussion disclosure but is currently unaddressed in concussion survey research. Many return-to-learn protocols recommend reduced screen time immediately post-concussion [47, 48, 49]. Might college age patients view this as a greater cost to self-disclosing than researchers suspect? There might be too many perceived specific costs to comprehensively ask about them in each survey, but a general cost-benefit approach might capture these effects while also allowing for easier translation between different populations.

The obtained evidence in favor generalized costs and rewards being fundamental explanations for cultures of non-disclosure should be interpreted in the context of study limitations. It is important to note that we measured the behavioral intention to disclose, rather than concussion-disclosing behavior. This use of a behavioral intention measure opens the possibility that individuals who were truly concussed might behave inconsistently with their reported intent to disclose. Nevertheless, we developed the concussion scenario because it provided notable advantages. It allowed us to include all participants rather than only those who reported concussions. It avoided the problems that can accompany retrospective accounts regarding why participants did or did not report concussions. It allowed us to obtain a large sample of participants which supported more expansive analyses than can be conducted in research with smaller sample sizes. We also believe that the anonymity of the survey provided a useful estimate of which cadets would disclose or fail to disclose their concussions thereby making our findings applicable to actual disclosure. Existing research supports this assumption [31]. We also suspect that the influence of perceived costs and rewards would be even stronger in terms of concussion-disclosing behavior because participants might have overestimated their willingness to disclose concussions in the face of perceived repercussions.

The present research was also non-experimental which introduces a need to consider issues related to causality. Fortunately, it is easy to argue that perceived costs and rewards influence concussion disclosure. Avoiding behaviors that are perceived to be costly is both intuitively obvious and supported by decades of psychological research [50]. Nevertheless, it is possible that other mechanisms could be at work, such as first developing a negative attitude about concussions and then inferring from that attitude that concussion disclosure must somehow be costly.

### Improving concussion reporting interventions

Despite these considerations, the present research suggests that researchers and practitioners would be most effective in improving concussion-disclosing behaviors by directly addressing the real and perceived costs that create concussion disclosure dilemmas. The common focus on identity and cultural factors is sensible, because these variables can contribute to concussion non-disclosure. Nevertheless, encouraging disclosure by addressing identity or social concerns can overlook the foundational concerns that cause concussion non-disclosure. Competitive athletes often believe that they have good reason to avoid concussion disclosure; they do not want to miss game time, to let down others, or to lose their scholarships. Likewise, future pilots at the Academy frequently believe that concussion disclosure could be costly, presumably because they worry that they will not be allowed to fly. Encouraging these populations to disclose concussions is unlikely to be effective if researchers and practitioners cannot address the underlying perceived cost-benefit structure. This possibly explains why educational initiatives have a negligible [51] or possibly negative influence on disclosure [52].

More effective interventions might assess the real and perceived costs of disclosure and work to mitigate the actual and perceived cost-benefit structure to make concussion disclosure more fundamentally appealing. This dilemma-focused approach can be implemented while also supporting concomitant culture change. For example, athletes who immediately report a concussion and stop playing have shorter recoveries [53] than those who delay. Explaining this finding to athletes might cause athletes to view concussion disclosure as less costly and encourage them to support disclosure in their respective communities. For USAFA pilots, the perception that concussion disclosure will undermine their careers appears to be largely unfounded; thus, practitioners might be able to encourage concussion disclosure by providing a more realistic of picture of the likely consequences of disclosure combined with examples of pilots who disclosed, recovered, and flew again. Concussion disclosure might also be broadly encouraged by making disclosure as easy as possible (e.g. short waiting times) and ensuring that individuals view disclosure as a method for facilitating recovery (a reward) rather than the source of delayed recovery (a cost) [54].

Similarly, policies that reduce the real cost of concussions could be effective, as long as those benefits are well-communicated to the sub-populations of concern. If a standard college concussion lasts 10 days and missing games is a primary concern for athletes, then a schedule offering more time between competitions could increase concussion reporting if athletes recognize that a concussion might cause them to miss a single game, rather than two games. Notably, this policy change might cause a concomitant cost of a longer athletic season. Likewise, if athletes and cadets conceal concussions for time management reasons, then return-to-learn programs that adjust schedules to ease post-concussion recoveries could provide an effective mitigation. These are just two examples of how practitioners could address costs to increase concussion disclosure. In short, through policy and outreach practitioners should attempt to make concussion self-disclosure more rewarding than costly and ensure that they communicate effectively about changes designed to encourage self-disclosure.

### Conclusion

Researchers interested in concussion self-disclosure have made tremendous advancements. In addition to highlighting concussion self-disclosure as an important public health problem, they have also identified several factors that contribute to non-disclosure. Indeed, the success that researchers have had in describing athlete concussion non-disclosure motivated us to expand this research into the military domain. We believe that our research supports this burgeoning literature by demonstrating that military members also report substantial concerns about concussion disclosure. Furthermore, considering concussion non-disclosure as both an athlete problem and an occupational problem requires one to consider generalized perceived costs and rewards as broad concepts that can explain concussion non-disclosure across populations. The obtained results support this perspective and suggest that researchers and practitioners must be attentive to the specific concerns that make concussion disclosure appear generally costly, even though the specific concerns might idiosyncratic to people and populations.
